# Two combinatorial optimization problems for SNP discovery using base-specific cleavage and mass spectrometry

**DOI:** 10.1186/1752-0509-6-S2-S5

**Published:** 2012-12-12

**Authors:** Xin Chen, Qiong Wu, Ruimin Sun, Louxin Zhang

**Affiliations:** 1 School of Physical and Mathematical Sciences, Nanyang Technological University, Singapore; 2 The Key Laboratory of Embedded System and Service Computing, Ministry of Education; Tongji University, Shanghai 200092, China; 3 Department of Mathematics, National University of Singapore, Singapore

## Abstract

**Background:**

The discovery of single-nucleotide polymorphisms (SNPs) has important implications in a variety of genetic studies on human diseases and biological functions. One valuable approach proposed for SNP discovery is based on base-specific cleavage and mass spectrometry. However, it is still very challenging to achieve the full potential of this SNP discovery approach.

**Results:**

In this study, we formulate two new combinatorial optimization problems. While both problems are aimed at reconstructing the sample sequence that would attain the minimum number of SNPs, they search over different candidate sequence spaces. The first problem, denoted as $\text{SNP - }\text{M}{\text{S}}_{P}$, limits its search to sequences whose *in silico *predicted mass spectra have all their signals contained in the measured mass spectra. In contrast, the second problem, denoted as $\text{SNP - M}{\text{S}}_{Q}$, limits its search to sequences whose *in silico *predicted mass spectra instead contain all the signals of the measured mass spectra. We present an exact dynamic programming algorithm for solving the $\text{SNP - }\text{M}{\text{S}}_{P}$ problem and also show that the $\text{SNP - M}{\text{S}}_{Q}$ problem is NP-hard by a reduction from a restricted variation of the 3-partition problem.

**Conclusions:**

We believe that an efficient solution to either problem above could offer a seamless integration of information in four complementary base-specific cleavage reactions, thereby improving the capability of the underlying biotechnology for sensitive and accurate SNP discovery.

## Background

Single nucleotide polymorphisms (SNPs) is a common type of DNA sequence variations that occur when a single nucleotide base is altered at a specific locus. They are among the most important genetic factors that contribute to human disease and biological functions. However, discovering novel SNPs is a scientifically challenging task. Among others, one valuable approach proposed for SNP discovery is based on base-specific cleavage and mass spectrometry [1-3].

The SNP discovery approach based on base-specific cleavage and mass spectrometry usually adopts a data-acquisition procedure as summarized below. First, a target sample DNA sequence is PCR-amplified using primers that incorporate the T7 promoter sequences. Then, the PCR products are in-vitro transcribed and subsequently digested with the endonuclease RNase A in four base-specific cleavage reactions. Each reaction can cleave the sample sequence to completion at all loci wherever a specific base is found. Finally, the matrix-assisted laser desorption/ionization time-of-flight mass spectrometry (MALDI-TOF MS) is applied to the cleavage products, resulting in four measured mass spectra, each corresponding to one base-specific cleavage reaction.

**Figure 1:**
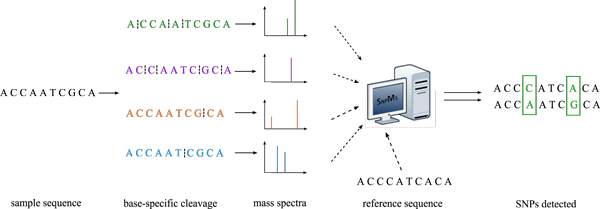
**Schematic outline**. The SNP discovery approach using base-specific cleavage and mass spectrometry.

Since each cleavage product is expected to be made of three non-cleavage bases, it is fairly straightforward to calculate the base composition from its measured mass signal. With all these base compositions in hand, the task of discovering SNPs in the sample sequence is now left to a computational solution. In principle, this computational solution shall find a way to integrate the four complementary base-specific mass spectra, and then identify those SNPs that necessarily account for the unanticipated base compositions (i.e., corresponding to the measured mass signal changes as compared with an *in-silico *predicted mass spectra from a reference sequence). See Figure 1 for schematic outline of the SNP discovery approach using base-specific cleavage and mass spectrometry.

The early proof-of-concept studies on the above SNP discovery approach using base-specific cleavage and mass spectrometry were presented in [3-5], where the identification of SNPs however was done by visual inspection. Shortly afterwards, two automated computational solutions were developed [1,2]: one was implemented in the proprietary MassARRAY™ SNP Discovery software package from Sequenom, Inc. and the other implemented in the software package called RNaseCut which is instead freely available online [6]. In particular, the solution in [1] mainly comprises of two separate procedures. It first computes all potential SNPs that give rise to each unanticipated based composition and then score them by taking into account the mass spectrometry data from the four base-specific cleavage reactions. Thus, the integration of the four base-specific cleavage reactions was done only in the second step. Apparently, such an integration strategy is far from being optimal, as at least it assumes that the occurrences of potential SNPs are independent in the first step.

In this paper, we study two new combinatorial optimization problems to exploit the full potential of the above SNP discovery approach. While both problems are aimed at reconstructing the sample sequence that would attain the minimum number of SNPs, they search over different candidate sequence spaces. The first problem, denoted as $\text{SNP - }\text{M}{\text{S}}_{P}$, limits its search to sequences whose *in silico *predicted mass spectra have all their signals contained in the measured mass spectra. In contrast, the second problem, denoted as $\text{SNP - M}{\text{S}}_{Q}$, limits its search to sequences whose *in silico *predicted mass spectra instead contain all the signals of the measured mass spectra. Then, we present an exact dynamic programming algorithm for solving the $\text{SNP - }\text{M}{\text{S}}_{P}$ problem and also show that the $\text{SNP - M}{\text{S}}_{Q}$ problem is NP-hard by a reduction from the restricted variation of the 3-partition problem [7,8].

## Methods

### Preliminaries

Let *s *∈ Σ* denote a string over the four-base alphabet ∑={A,C,G,T}. The length of *s *is denoted by |*s*|, the *i*-th base of *s *by *s*[*i*], and the substring of *s *from the *i*-th base to the *j*-th base by *s*[*i*, *j*], for 1 ≤ *i *≤ *j *≤ |*s*|. We use *∈ *to denote the empty string so that |*∈| *= 0. The concatenation of two strings *s *and *t *is denoted by *s · t*, and the concatenation of *l *copies of a string *s *is denoted by *s^l^*.

Given a string *s *and a cut base x∈∑, a *cleavage fragment *refers to a substring of *s *that does not contain *x *and that cannot be extended in either side without crossing a base *x*. Formally, the substring *s*[*i*, *j*] is a cleavage fragment with respect to the cut base *x *if the following three conditions are satisfied: (i) *s*[*i − *1] = *x *if *i *≠ 1, (ii) *s*[*j *+ 1] = *x *if *j *≠ |*s*|, and (iii) *s*[*k*] ≠ *x*, ∀*k *∈ [*i*, *j*]. In addition, the empty string *ε *is a cleavage fragment if there exits *i *∈ [1,|*s| − *1] such that *s*[*i*] = *s*[*i *+ 1] = *x*. Given a cleavage fragment, we use ${\text{A}}_{i}{\text{C}}_{j}{\text{G}}_{k}{\text{T}}_{l}$ to denote its base composition of *i *As, *j *Cs, *k *Gs, and *l *Ts. In [1], this base composition is termed as a *compomer *of the string *s *with respect to the cut base *x*. The whole set of compomers is hence called the *compomer spectrum *of the string *s *with respect to the cut base *x*, and denoted by Finally, let $C_{\sum }\left(s\right)=\left\{C_{x}\left(s\right):x\in \sum \right\}=\left\{C_{\text{A}}\left(s\right),C_{\text{C}}\left(s\right),C_{\text{G}}\left(s\right),C_{\text{T}}\left(s\right)\right\}$, a collection of four compomer spectra of the string *s *where each is generated with one cut base.

**Example 1 ***Let s *:= ACATGCTACATTA. *Then, the string s contains four cleavage fragments with respect to the cut base *A*: *C, TGCT, C, *and *TT. *With respect to the cut base *T, *it instead contains five cleavage fragments: *ACA, GC, ACA, *∈, and *A. *Their respective compomer spectra are $C_{\text{A}}\left(s\right)=\left\{{\text{A}}_{0}{\text{C}}_{\text{1}}{\text{G}}_{0}{\text{T}}_{0},{\text{A}}_{0}{\text{C}}_{\text{1}}{\text{G}}_{\text{1}}{\text{T}}_{\text{2}},{\text{A}}_{0}{\text{C}}_{0}{\text{G}}_{0}{\text{T}}_{\text{2}}\right\}$ and $C_{\text{T}}\left(s\right)=\left\{{\text{A}}_{\text{2}}{\text{C}}_{\text{1}}{\text{G}}_{0}{\text{T}}_{0},{\text{A}}_{0}{\text{C}}_{\text{1}}{\text{G}}_{\text{1}}{\text{T}}_{0},{\text{A}}_{0}{\text{C}}_{0}{\text{G}}_{0}{\text{T}}_{0},{\text{A}}_{\text{1}}{\text{C}}_{0}{\text{G}}_{0}{\text{T}}_{0}\right\}$. Note that each compomer appears in a compomer spectrum at most once*.

### Problem formulation

Let *d_H _*$\left(s,s^{\prime }\right)$ denote the Hamming distance between two strings *s *and $s^{\prime }$ of equal length. It measures the minimum number of substitutions required to transform one string into the other. Given a collection of compomer spectra $C_{\Sigma }=\left\{C_{x}:x\in \Sigma \right\}$ of an unknown string $s^{\prime }$ (i.e., the sample DNA sequence experimented) which can in principle be generated from a mass spectrometry experiment, and a string *s *(i.e., the reference DNA sequence) which is believed to differ from the unknown string $s^{\prime }$ by a number of substitutions only, we formulate below two combinatorial optimization problems for SNP discovery.

**Definition 2 **$\left(The\;SNP-MS_{P}\;problem\right)$*Given a string s and a collection of compomer spectra *$C_{\Sigma }=\left\{C_{x}:x\in \Sigma \right\}$, *find a string *$s^{\prime }$*such that $C_{x}\left(s^{\prime }\right)\subseteq C_{x}$, for all x∈∑ and d_H _*$\left(s,s^{\prime }\right)$*is minimized*.

**Definition 3 **$\left(The\;SNP-MS_{Q}\;problem\right)$*Given a string s and a collection of compomer spectra *$C_{\Sigma }=\left\{C_{x}:x\in \Sigma \right\}$, *find a string *$s^{\prime }$*such that $C_{x}\subseteq C_{x}\left(s^{\prime }\right)$, for all *x∈∑*and d_H _*$\left(s,s^{\prime }\right)$*is minimized*.

The only difference between the above two problem formulations is that one requires $C_{x}\left(s^{\prime }\right)\subseteq C_{x}$ and the other requires $C_{x}\subseteq C_{x}\left(s^{\prime }\right)$, for all the cut bases. Once the string $s^{\prime }$ is found, it is easy to identify the SNPs in $s^{\prime }$, i.e., those base substitutions that transform $s^{\prime }$ into *s*.

**Example 4 ***In this example, we let *∑:={A,T}*for simplicity. Given the string s*:= ATAAT *and the set *$C=\left\{C_{\text{A}},C_{\text{T}}\right\}$*of compomer spectra (of an unknown string) where*

$$C_{\text{A}}=\left\{{\text{A}}_{0}{\text{T}}_{1},{\text{A}}_{0}{\text{T}}_{2}\right\}\;\text{and}\;C_{\text{T}}=\left\{{\text{A}}_{0}{\text{T}}_{0},{\text{A}}_{1}{\text{T}}_{0}\right\}.$$

*The feasible solutions to the *$\text{SNP - }\text{M}{\text{S}}_{P}$*problem for the above instance include the strings such as *ATATA, TATAT, TTATT, ATATT, *and *ATTAT. *Their respective Hamming distances to the input string s are 2*, *3, 2, 1, and 1. The string *$s^{\prime }$ = TTAAT *is not a feasible solution because the compomer ${\text{A}}_{\text{2}}{\text{T}}_{0}\in C_{\text{T}}\left(s^{\prime }\right)$ but ${\text{A}}_{\text{2}}{\text{T}}_{0}\notin C_{\text{T}}$ so that $C_{\text{T}}\left(s^{\prime }\right)⊈C_{\text{T}}$*.

*The feasible solutions to the $SNP-MS_{Q}$ problem for the above instance include the strings such as *TTATA, TATTA, ATATT, *and *ATTAT. *Their respective Hamming distances to the input string s are 3, 5, 1, and 1*. *The string *$s^{\prime }$ = TTAAT *is not a feasible solution because the compomer ${\text{A}}_{\text{2}}{\text{T}}_{0}\in C_{\text{T}}$ but ${\text{A}}_{\text{1}}{\text{T}}_{0}\notin C_{\text{T}}\left(s^{\prime }\right)$ so that $C_{\text{T}}⊈C_{\text{T}}\left(s^{\prime }\right)$*.

The measured mass spectra of a sample sequence are rarely perfect in practice. Some peaks may actually represent noises, while some true signal peaks are missing. The problem $\text{SNP - }\text{M}{\text{S}}_{P}$ is so formulated that its computational solution would be robust against noisy peaks but susceptible to missing peaks (i.e., there is a good chance to recover the sample sequence even if some noisy peaks are present in the measured mass spectra, but the chance would become much less if there are some true signal peaks missing). In contrast, the problem $\text{SNP - M}{\text{S}}_{Q}$ is so formulated that its computational solution would be robust against missing peaks but susceptible to noisy peaks.

We noticed that several computational problems in the literature that are more or less related to our problems introduced above. In [9], a so-called *sequencing from compomers *problem was studied which, like the $\text{SNP - }\text{M}{\text{S}}_{P}$ problem, also aimed to reconstruct the sample sequence from a given collection of compomer spectra, but without help of a reference sequence. In [10], the *spectral alignment *problem differs from the $\text{SNP - }\text{M}{\text{S}}_{P}$ problem mainly by its exploration on short read sequencing data rather than the mass/compomer spectra data, which may lead to wide implications in the subsequent algorithm design and complexity analysis. Moreover, in [1], a so-called *SNP discovery from mass spectrometry *problem was defined in a similar way to the $\text{SNP - M}{\text{S}}_{Q}$ problem. However, it has only a single compomer as input, as opposed to a collection of four complementary compomer spectra used in the $\text{SNP - M}{\text{S}}_{Q}$ problem.

## Results

### An exact dynamic programming algorithm for $\text{SNP - }\text{M}{\text{S}}_{P}$

In this subsection, we shall describe an exact dynamic programming algorithm for solving the $\text{SNP - }\text{M}{\text{S}}_{P}$ problem. Without loss of generality, we may assume in the remaining of this section that every base of Σ will eventually occur in the optimal solution to a given instance of the $\text{SNP - }\text{M}{\text{S}}_{P}$ problem. Consequently, only those feasible solutions that contains all the bases of Σ need to be considered when we search for the optimal solution. In case some base *x *would not occur in the optimal solution $s^{\prime }$ note that it becomes relatively easy to find $s^{\prime }$ since we would have $s^{\prime }\in L_{x}\cap R_{x}$ and |*s*'| = |*s*|. See below for definitions of $L_{x}$ and $R_{x}$.

Let us start with some preliminary definitions and notations. For a string *s*, a cleavage fragment *s*[*i*, *j*] is called *internal *if neither *i *= 1 nor *j *= |*s*|, *left-ended *if *i *= 1, or *right-ended *if *j *= |*s*|. In addition, a cleavage fragment *∈ *is always considered internal. Given a collection of compomer spectra $C_{\sum }$, we call a string is *I-compatible *if the compomers of its internal cleavage fragments are all contained in $C_{\sum }$ (under the respective cut base). A string is called *L-compatible *(resp. *R-compatible*) if it is I-compatible and if the compomers of its left-ended (resp. right-ended) cleavage fragments are all contained in $C_{\sum }$ as well.

**Example 5 ***Consider the string s given in Example 1. The four cleavage fragments of s with respect to the cut base *A *are all internal. Among the five cleavage fragments of s with respect to the base *T, *the first cleavage fragment *ACA *is left-ended, the last cleavage fragment *A *is right-ended, and the other three cleavage fragments in the middle are all internal*.

**Example 6 ***Let *$C_{\sum }=\left\{C_{\text{A}},C_{\text{C}},C_{\text{G}},C_{\text{T}}\right\}$*be a collection of compomer spectra where*

$$\begin{array}{c} C_{\text{A}}=\left\{{\text{A}}_{0}{\text{C}}_{1}{\text{G}}_{0}{\text{T}}_{0},{\text{A}}_{0}{\text{C}}_{1}{\text{G}}_{1}{\text{T}}_{2},{\text{A}}_{0}{\text{C}}_{0}{\text{G}}_{0}{\text{T}}_{2}\right\},\\ C_{\text{C}}=\left\{{\text{A}}_{1}{\text{C}}_{0}{\text{G}}_{0}{\text{T}}_{0},{\text{A}}_{1}{\text{C}}_{0}{\text{G}}_{1}{\text{T}}_{1},{\text{A}}_{1}{\text{C}}_{0}{\text{G}}_{0}{\text{T}}_{1},{\text{A}}_{2}{\text{C}}_{0}{\text{G}}_{0}{\text{T}}_{2}\right\},\\ C_{\text{G}}=\left\{{\text{A}}_{2}{\text{C}}_{1}{\text{G}}_{0}{\text{T}}_{1},{\text{A}}_{3}{\text{C}}_{2}{\text{G}}_{0}{\text{T}}_{3}\right\},\;\text{and}\\ C_{\text{T}}=\left\{{\text{A}}_{2}{\text{C}}_{1}{\text{G}}_{0}{\text{T}}_{0},{\text{A}}_{0}{\text{C}}_{1}{\text{G}}_{1}{\text{T}}_{0},{\text{A}}_{0}{\text{C}}_{0}{\text{G}}_{0}{\text{T}}_{0},{\text{A}}_{1}{\text{C}}_{0}{\text{G}}_{0}{\text{T}}_{0}\right\}. \end{array}$$

*We show in *Table 1* whether each of the given strings is I-compatible, L-compatible, or R-compatible with *$C_{\sum }$.

**Table 1:** Examples.

strings	I-compatible	L-compatible	R-compatible
ATGATAC			
ATGCTAC			
ACATGCT			
TACATTA			
CTACATTA			

This table shows whether each of the given strings is I-compatible, L-compatible, or R-compatible with $C_{\sum }$.

For each compomer ${\text{A}}_{i}{\text{C}}_{j}{\text{G}}_{k}{\text{T}}_{l}\in C_{x}$ in a given collection of compomer spectra $C_{\sum }$, we use $I_{x}\left({\text{A}}_{i}{\text{C}}_{j}{\text{G}}_{k}{\text{T}}_{l}\right)$ to denote the set of strings that (i) consist of *i *As, *j *Cs, *k *Gs, *l *Ts, (ii) contain exactly three distinct bases (i.e., three bases in the set Σ *\ *{*x*}), and (iii) are I-compatible with $C_{\sum }$. It is easy to check that $|I_{x}\left({\text{A}}_{i}{\text{C}}_{j}{\text{G}}_{k}{\text{T}}_{l}\right)|\;\leq \frac{\left(i+j+k+l\right)!}{i!j!k!l!}$. In particular, if there exists in A_*i*_C_*j *_G_*k*_T_*l *_a non-cut base whose composition value is zero, then we have $I_{x}\left({\text{A}}_{i}{\text{C}}_{j}{\text{G}}_{k}{\text{T}}_{l}\right)\;=\emptyset$ so that $|I_{x}\left({\text{A}}_{i}{\text{C}}_{j}{\text{G}}_{k}{\text{T}}_{l}\right)|\;=0$. Furthermore, we may define the following set

$$I_{x}=\underset{{\text{A}}_{i}{\text{C}}_{j}{\text{G}}_{k}{\text{T}}_{l}\in C_{x}}{⋃}I_{x}\left({\text{A}}_{i}{\text{C}}_{j}{\text{G}}_{k}{\text{T}}_{l}\right),\;\forall x\in \Sigma .$$

Then, let $I_{\Sigma }=\left\{I_{\text{A}},\;I_{\text{C}},\;I_{\text{G}},\;I_{\text{T}}\right\}$. Analogously, we may define $L_{x}\left({\text{A}}_{i}{\text{C}}_{j}{\text{G}}_{k}{\text{T}}_{l}\right)$, $R_{x}\left({\text{A}}_{i}{\text{C}}_{j}{\text{G}}_{k}{\text{T}}_{l}\right)$, $L_{\Sigma }=\left\{L_{\text{A}},\;L_{\text{C}},\;L_{\text{G}},\;L_{\text{T}}\right\}$ and *$R_{\Sigma }=\left\{R_{\text{A}},\;R_{\text{C}},\;R_{\text{G}},\;R_{\text{T}}\right\}$*for the L-compatible strings and the R-compatible strings, respectively. Clearly, $L_{x}\subseteq I_{x}$ and $R_{x}\subseteq I_{x}$, for all *x *∈ Σ.

**Example 7 ***Consider the collection of compomer spectra *$C_{\sum }$*given in Example 6. For the compomer *${\text{A}}_{0}{\text{C}}_{\text{1}}{\text{G}}_{\text{1}}{\text{T}}_{\text{2}}\in C_{\text{A}}$, *we have *$I_{\text{A}}\left({\text{A}}_{0}{\text{C}}_{\text{1}}{\text{G}}_{\text{1}}{\text{T}}_{\text{2}}\right)\;=\left\{\text{CGTT},\text{CTTG},\text{GCTT},\text{GTTC},\text{TCGT},\text{TGCT},\text{TTCG},\text{TTGC}\right\}$, *and *$L_{\text{A}}\left({\text{A}}_{0}{\text{C}}_{1}{\text{G}}_{1}{\text{T}}_{2}\right)=R_{\text{A}}\left({\text{A}}_{0}{\text{C}}_{1}{\text{G}}_{1}{\text{T}}_{2}\right)=\emptyset$. For the compomer ${\text{A}}_{0}{\text{C}}_{\text{1}}{\text{G}}_{\text{1}}{\text{T}}_{0}\in C_{\text{T}}$, *we have *$I_{\text{T}}\left({\text{A}}_{0}{\text{C}}_{1}{\text{G}}_{1}{\text{T}}_{0}\right)=L_{\text{T}}\left({\text{A}}_{0}{\text{C}}_{1}{\text{G}}_{1}{\text{T}}_{0}\right)=R_{\text{T}}\left({\text{A}}_{0}{\text{C}}_{1}{\text{G}}_{1}{\text{T}}_{0}\right)=\emptyset$.

Given a string *t *which could be a potential cleavage fragment with respect to the cut base *x *(i.e., the string *t *does not contain any base *x*), we say a string *s *begins with the string *t *if *t · x *is a prefix of *s · x*, or say a string *s *ends with the string *t *if *x · t *is the suffix of *x · s*. The following lemma is useful to design a dynamic programming algorithm for solving the $\text{SNP - }\text{M}{\text{S}}_{P}$ problem. Its easy proof is omitted. Recall that our discussions in this section are limited only to the feasible solutions containing all the bases of Σ.

**Lemma 8 ***A string s*' *of length* |*s| is a feasible solution to the *$\text{SNP - }\text{M}{\text{S}}_{P}$*problem if and only if*

*- all the substrings of *$s^{\prime }$*are I-compatible with *$C_{\sum }$,

*- *$s^{\prime }$*begins with a string in $L_{x}$ for some *x∈∑, *and*

*- *$s^{\prime }$*ends with a string in $R_{x}$ for some*x∈∑.

Suppose we have an input instance $\left\langle s,C_{\sum }\right\rangle$ of the $\text{SNP - }\text{M}{\text{S}}_{P}$ problem. Given a string $t\in I_{x}$ where x∈∑, we define $H\left(i,\;t\right)$ to be the minimum Hamming distance between the prefix of *s *of length *i *and a string which is such that

- all its substrings are I-compatible with $C_{\sum }$,

- it begins with a string from $L_{y}$ for some *y *∈ Σ, and

- it ends with the given string *t*.

To compute $H\left(i,\;t\right)$, we first find in the string *x · t *the rightmost position *k *at which the base (*x · t*)[*k*] is its first occurrence. Formally, we may write

k=max{j:∀i,1≤i<j≤|x⋅t|,(x⋅t)[i]≠(x⋅t)[j]}.

Then, let *x*':= (*x *· *t*)[*k*], *p *:= (*x *· *t*)[1, *k *- 1], and *q *:= (*x *· *t*)[*k*,| *x *· *t*|]. Note that *x*' ≠ *x *and the string *p *contains all the bases of Σ except *x*'.

**Example 9 ***Let t *:= CGTT **∈ ***I*_A_. *Then*, *x · t *= ACGTT, *k *= 4, $x^{\prime }=\text{T}$, *p *= ACG, *and q *= TT.

To compute H(i,t), we now use the following recurrence relation

$$H\left(i,\;t\right)=\;\underset{t^{\prime }\in I_{x^{\prime }}}{\text{min}}\;\left\{H\left(i-|q|,\;t^{\prime }\right)+d_{H}\left(s\left[i-|q|+1,\;i\right],\;q\right)\right\}.$$

$$\exists t^{\prime \prime },t^{\prime }=t^{\prime \prime }\cdot p$$

Note that the minimization in the above is taken over all those strings $t^{\prime }$*^′ ^*in $I_{x^{\prime }}$ which have *p *as the suffix. If there is no such a string in $I_{x^{\prime }}$, then we let H(i,t)=∞. The initial conditions for the recurrence relation are given as follows:

$$H\left(i,\;t\right)=\left\{\begin{array}{cc} \infty & \text{if}\;i<|t|\;\text{and}\;t\in I_{x}\\ d_{H}\left(s\left[1,\;i\right],\;t\right) & \text{if}\;i=|t|\;\text{and}\;t\in L_{x}\\ \infty & \text{if}\;i=|t|\;\text{and}\;t\in I_{x}\backslash L_{x}. \end{array}\right)$$

**Theorem 10 ***Let s*' *be the string that leads to*

$$d_{H}\left(s,\;s^{\prime }\right)=\;\underset{\forall t\in R_{x},x\in \Sigma }{\text{min}}H\left(|s|,\;t\right),$$

*then s*' *would be an optimal solution to the input instance *$\left\langle s,C_{\sum }\right\rangle$*of the *$SNP\text{ - }MS_{P}$*problem*.

*Proof: *For the correctness of the above dynamic programming algorithm, we need to show that (i) every feasible solution of the $\text{SNP - }\text{M}{\text{S}}_{P}$ problem would be essentially evaluated by the dynamic programming algorithm, and (ii) every string evaluated by the dynamic programming algorithm must be a feasible solution of the $\text{SNP - }\text{M}{\text{S}}_{P}$ problem.

Let the string *s*' be a feasible solution. Consider a cleavage fragment *t *of *s*' that contains all the bases of Σ except its corresponding cut base *x*. Clearly, $t\in I_{x}$ and *t *is the suffix of a substring *s*'[1, *i*] for some integer *i*. Without loss of generality, we can further suppose that *t *≠ *s*'[1, *i*]. To show (i), what we mainly need to show is that there exists a string $t^{\prime }\in I_{x^{\prime }}$ such that *p *is the suffix of *t*' and *t*' is the suffix of the substring *s*'[1, *i - *|*q*|], where *x*', *p*, and *q *are computed for the string *t *as described earlier. Indeed, we can find the string *t^′ ^*as follows. First, let (*i*' *− *1) be the position of the last occurrence of the base *x*' in the substring *s*'[1, *i − |t*|]; if there is no such occurrence, we let *i*' = 1. Then, we assign $t^{\prime }:=s^{\prime }\left[i^{\prime },i-|q|\right]$. Obviously, $t^{\prime }$ is the suffix of *s*'[1, *i *|*q*|]. Because *s*'[*i - *|*t*|] = *x *and *x *≠ *x *, we have *i*' ≤ *i - |t*|. It then follows from *p *= *s*'[*i − |t|, i − |q*|] that *p *shall be the suffix of *t*'. Since *p *contains all the bases of  ∑ except *x*' so, does *t*'. Moreover, *t*' is a cleavage fragment of *s*' with respect to the cut base *x*' because we have either *s*'[*i*' *− *1] = *x*' or *i*' = 1 on the left end of *t*' and *s*'[*i − |q| *+ 1] = *x*' on the right end of $t^{\prime }$. By Lemma 8, we can see that $t^{\prime }\in I_{A}$. For the reader's convenience, we demonstrate in the following example how to find $t^{\prime }$ from *t*. Let *s*' = ACATGCTACATTA, *t *= *s*' [4,7] = TGCT, *i *= 7, *x *= A, and $C_{\sum }$ be the one as given in Example 6. Note that$t\in I_{A}$. Further, for the given string *t *= TGCT, we have *x*' = C, *p *= ATG, and *q *= CT. Then, we obtain that *i*' = 3 and then *t*' = *s*' [3, 7 *− *2] = *s*' [3,5] = ATG. It is easy to check that *p *is the suffix of *t*', *t*' is the suffix of the substring $s^{\prime }\left[1,i-|q|\right]$, and $t^{\prime }\in I_{x^{\prime }}$.

On the other hand, let *s*' be a string evaluated by the dynamic programming algorithm. So, the string *s*' must begin with a string in $L_{x}$ for some x∈∑ and end with a string in $R_{y}$ for some y∈∑. Consider a cleavage fragment *t *of *s*' that was used to construct the string *s*' during the backtracking procedure of the algorithm. Clearly, the string *t *contains all the bases of  ∑ except its corresponding cut base *x*. Moreover, $t\in I_{x}$ and *t *is the suffix of a substring *s*'[1, *i*] for some integer *i*. Without loss of generality, we can further suppose *t *≠ *s*'[1, *i*] and $i\neq |s^{\prime }|$, so that *s*'[*i − |t*|] = *s*'[*i *+ 1] = *x*. Let *t*' be the string considered next to the string *t *during the backtracking procedure of the algorithm. Thus, we have $t^{\prime }\in I_{x^{\prime }}$ such that *p *is the suffix of *t*' and $t^{\prime }$ is the suffix of the substring *s*'[1, *i − |q*|], where *x*', *p*, and *q *are computed for the string *t *as described earlier. More specifically, there exists *i*' such that *t' *= *s*'[*i*', *i −|q*|] and *s^′^*[*i*' *−*1] = *s*' [*i −|q| *+ 1] = *x*' if *i*' ≠ 1. To show (ii), by Lemma 8 and also by backward induction, what we mainly need to show is that the extended substring *s*'[*i*'*,|s*'|] is I-compatible with $C_{\sum }$, given that the substring *s*'[*i − |t| *+ 1, |*s*'|] is already I-compatible with $C_{\sum }$. To this end, we consider any internal cleavage fragment *s*'[*j*, *k*] of *s*' [*i*', |*s*'|] with respect to the cut base *x″ *= *s*'[*j − *1] = *s*'[*k *+ 1]. By definition of the internal cleavage fragment, we have *j *≥ *i*' + 1 and *k *≤ |*s*'| *− *1. In the following we distinguish four cases:

- If *j *≥ *i − |t| *+ 2, then *s*'[*j*, *k*] is an internal cleavage fragment of *s*'[*i − |t| *+1, |*s*'|]. Since *s*'[*i − |t| *+1, |*s*'|] is already assumed to be I-compatible with $C_{\sum }$, the base composition of *s*'[ *j*, *k*] shall be also contained in $C_{x^{\prime \prime }}$.

- If *j *= *i − |t| *+ 1, then *x*″ = *x*, which further implies that *k *= *i *and *s*' [*j*, *k*] = *t*. Since $t\in I_{x}$, the base composition of *s*'[*j*, *k*] shall be contained in $C_{x^{\prime \prime }}$.

- If *j *≤ *i − |t| *and *k *≥ *i − |q*|, then *s*'[*i − |t*|, *i − |q*|] is a substring of *s*'[*j*, *k*]. Since *s^′^*[*i − |t*|, *i − |q*|] contains all the bases of Σ, the string *s*'[*j*, *k*] can not be a cleavage fragment (as a cleavage fragment must not contain its corresponding cut base). Therefore, there shall not have the case where *j *≤ *i − |t| *and *k *≥ *i − |q*|.

- If *k *≤ *i − |q| − *1, then *s*'[*j*, *k*] is an internal cleavage fragment of *t*' = *s*'[*i*', *i − |q*|]. Since $t\in I_{x^{\prime }}$, the base composition of *s*'[*j*, *k*] shall be contained in $C_{x^{\prime \prime }}$.

In conclusion, for every internal cleavage fragment of *s*'[*i^′^*, |*s^′^*|], its base composition is contained in $C_{\sum }$ under the respective cut base. Therefore, the extended substring *s*'[*i*', |*s*'|] is still I-compatible with $C_{\sum }$.

Note that computing each entry H(i,t) of the dynamic programming table may take time $O\left(\left|s\right|\;\cdot |I_{\sum }|\right)$, where $\left|I_{\sum }|\;=\;|I_{\text{A}}|+|I_{\text{C}}|+|I_{\text{G}}|+|I_{\text{T}}\right|$. Hence, the above dynamic programming algorithm can be done in time $O\left(|s{|}^{\text{2}}\cdot |I_{\sum }{|}^{\text{2}}\right)$. In the worst case, we may have $|I_{\sum }|=O\left(|s|!\right)$, that is, $\left|I_{\sum }\right|$ is in the factorial order of the input problem size. In practice, however, we would expect $\left|I_{\sum }\right|$ not too large to be manageable, because cleavage fragments are usually of small size. Therefore, the above dynamic programming algorithm could be a practically feasible solution to the problem $\text{SNP - }\text{M}{\text{S}}_{P}$, especially when compared to the brute-force algorithm which needs to examine all the possible strings *s*'. For the special case where |∑|=2, $\text{SNP - }\text{M}{\text{S}}_{P}$ is actually an easy problem, as we can see from the above that $|I_{\sum }|\;=O\left(\left|s\right|\right).$

**Corollary 11 ***The above dynamic programming algorithm can solve the *$SNP-MS_{P}$ problem in polynomial time when |∑|=2.

### The NP-hardness of $\text{SNP - M}{\text{S}}_{Q}$

This subsection is dedicated to prove that the $\text{SNP - M}{\text{S}}_{Q}$ problem is NP-hard. We begin with a brief introduction of the 3-partition problem.

**Definition 12 (The general form of the 3-partition problem) ***Given a multiset of positive integers *$A=\left\{a_{1},\;a_{2},\;\cdots \;,\;a_{n}\right\}$*where n *= 3*m and $\sum_{i=1}^{n}a_{i}=mB$, can we partition the multiset A  into m multisets *$A_{1},A_{2},\cdots \;,A_{m}$, *such that the sum of each multiset is equal to B?*

The 3-partition problem is strongly NP-complete [7]. Therefore, it remains NP-complete even when the integers in A  and the integer *B *are encoded in unary. In this case, the size of a problem instance is Θ(*nB*). In contrast, it becomes *O*(*n *log *B*) when using the binary encoding of integers.

**Definition 13 (The restricted variation of the 3-partition problem) ***Given a set of positive integers *$A=\left\{a_{1},\;a_{2},\;\cdots \;,\;a_{n}\right\}$*where n *= 3*m, $\sum_{i=1}^{n}a_{i}=mB$, and $\frac{B}{4}<a_{i}<\frac{B}{2},\forall 1\leq i\leq n$, can we partition the set *A *into m subsets *$A_{1},A_{2},\cdots \;,A_{m}$, *such that the sum of each subset is equal to B?*

There are two constraints imposed in the above restricted variation of the 3-partition problem. The first one limits A  to be a set so that all the integers in A  are distinct. The second one limits all the integers in A  strictly between $\frac{B}{4}$ and $\frac{B}{2}$, which subsequently enforces every subset $A_{i}$ to consist of exactly three elements. Interestingly, this restricted variation of the 3-partition problem remains strongly NP-complete [8], just like the general form of the 3-partition problem. Note that the second constraint $\frac{B}{4}<a_{i}<\frac{B}{2}$ was actually not imposed in [8]. But, it can be easily done by adding *B *to each *a_i _*and then multiplying *B *by 4.

**Theorem 14 ***The *$SNP-\;MS_{Q}$*problem is NP-hard, even when *|∑|=2.

*Proof: *We prove it by a reduction from the above restricted variation of the 3-partition problem. As an input for 3-partition, we are given a set of distinct positive integers $A=\left\{a_{1},\;a_{2},\;\cdots \;,\;a_{n}\right\}$ where *n *= 3*m*, $\sum_{i=1}^{n}a_{i}=mB$, and $\frac{B}{4}<a_{i}<\frac{B}{2},\forall 1\leq i\leq n$. Then, we construct an instance $\left\langle s,C_{\sum }\right\rangle$ of the $\text{SNP - M}{\text{S}}_{Q}$ problem as follows:

- Let Σ = {G, T}.

- Let *s *be the string such that *s · *T = (G^*B*+2^T)*^m^*. That is, let *s · *T be the concatenation of *m *copies of the fragment GG *· · · *GT, where each fragment consists of (*B *+ 2) consecutive base Gs followed by one base T. Note that |*s| *= *m*(*B *+ 3) *− *1 = *mB *+ 3*m − *1.

- Let $C_{\text{G}}=\left\{{\text{G}}_{0}{\text{T}}_{0},{\text{G}}_{0}{\text{T}}_{\text{1}}\right\}$ and $C_{\text{T}}=\left\{{\text{G}}_{a_{i}}{\text{T}}_{0}:\text{ 1}\leq i\leq n\right\}$ so that $C_{\sum }=\left\{C_{\text{G}},C_{\text{T}}\right\}$.

First, we check whether this construction can be done in polynomial time in the size of the input instance of the 3-partition problem. Since the restricted variation of the 3-partition problem is strongly NP-complete, we may encode the integers in unary so that the size of the input instance is Θ(*nB*). In the above reduction, we can easily see that the first step can be done in constant time, the second step in time *O*(*mB*), and the third step in time *O*(*n *log *B*). Therefore, the total time needed for construction is *O*(*nB*), no more than time polynomial in the size of the input instance of the 3-partition problem.

Next, we show that every feasible solution *s″ *to the reduced instance $\left\langle s,C_{\sum }\right\rangle$ of the $\text{SNP - M}{\text{S}}_{Q}$ problem is such that (i) $C_{\text{T}}\left(s^{\prime \prime }\right)=C_{\text{T}}$, (ii) *s*″ contains exactly 3*m − *1 base Ts, and (iii) *d_H _*(*s*, *s*″) *≥ *2*m*. For each compomer ${\text{G}}_{a_{i}}{\text{T}}_{0}\in C_{\text{T}}\subseteq C_{\text{T}}\left(s^{\prime \prime }\right)$, there exists at least one cleavage fragment $G^{a_{i}}$ in *s*″ that is obtained with respect to the cut base T. Since all the integers *a*_*i *_are distinct, all such cleavage fragments shall be pairwise non-overlapping. Thus, the string *s*′′ contains at least $\sum_{i=1}^{n}a_{i}=mB$ base Gs and at least *n *− 1 = 3*m - *1 base Ts. On the other hand, since |*s| *= *mB *+ 3*m - *1, the string *s*″ hence consists of exactly *mB *+ 3*m− *1 bases. Therefore, we can deduce that *s″ *contains exactly 3*m − *1 base Ts and further that $C_{\text{T}}\left(s^{\prime \prime }\right)$ cannot have any other compomer than those in *C*_T_. By construction, we also know that the string *s *contains exactly *m − *1 base Ts, which hence implies that *d*_*H *_(*s*, *s*″) ≥ 2*m*.

Now, we are going to show that there exists a valid partition for the input instance of the 3-partition problem if and only if there exists an optimal solution *s^′ ^*for the reduced instance of the $\text{SNP - M}{\text{S}}_{Q}$ problem such that *d*_*H *_(*s*, *s*') = 2*m*.

Suppose that A  can be partitioned into *m *subsets$A_{1},A_{2},\cdots \;,A_{m}$ such that, for each subset $A_{i}=\left\{a_{i_{1}},\;a_{i_{2}},\;a_{i_{3}}\right\}$, its size is three and its integer elements adds up to exactly *B*, that is, *$\left|A_{i}=3\right|$*and $\sum_{j=1}^{3}a_{i_{j}}=B,\forall 1\leq i\leq m$. Then, we use the following procedure to find the string *s*':

1.    $s^{\prime }:=\emptyset$;

2.    **for ***i *= 1 to *m*

3.      **for ***j *= 1 to 3

4.       $s^{\prime }+={\text{G}}^{a_{i_{j}}}\text{T}$; // append the string ${\text{G}}^{a_{i_{j}}}\text{T}$ to *s*'

5.      **end**

6.    **end**

7.    *s*':= *s*'[1, |*s*'| *− *1]; // remove the last base T

As one can easily check, the resulting string *s*' is such that |*s*'| = *mB *+ 3*m − *1, $C_{\text{G}}\subseteq C_{\text{G}}\left(s^{\prime }\right)$, and $C_{\text{T}}\subseteq C_{\text{T}}\left(s^{\prime }\right)$. Therefore, *s*' is a feasible solution to the reduced instance $\left\langle s,C_{\sum }\right\rangle$ of the $\text{SNP - M}{\text{S}}_{Q}$ problem. On the other hand, since $\sum_{j=1}^{3}a_{i_{j}}=B,\forall 1\leq i\leq m$, we can deduce that *s*'[*k*] = *s*[*k*] if *s*'[*k*] = G or *s*[*k*] = T; otherwise, *s^′^*[*k*] ≠ *s*[*k*], ∀*k *∈ [1, *mB *+ 3*m *- 1]. Therefore, *d*_*H *_(*s*, *s*') =|[*k *: *s*'[*k*] ≠ *s*[*k*]}| = |*s| − *|{*k *: *s*'[*k*] = *s*[*k*]}| = *mB *+ 3*m − *1 *− *|{*k *: *s*'[*k*] = G}| *− *|{*k *: *s*[*k*] = T}| = *mB *+ 3*m − *1 *− mB − m *+ 1 = 2*m*. It hence follows that *s′ *is indeed an optimal solution to the reduced instance $\left\langle s,C_{\sum }\right\rangle$ of the $\text{SNP - M}{\text{S}}_{Q}$ problem.

Conversely, suppose that the string *s*' is an optimal solution to the reduced instance $\left\langle s,C_{\sum }\right\rangle$ of the $\text{SNP - M}{\text{S}}_{Q}$ problem such that *d*_*H*_(*s*, *s*') = 2*m*. Then, we use the following procedure to find a partition $A_{1},A_{2},\cdots \;,A_{m}$ of *A*:

1.    *s *:= *s · *T; *s*':= *s*' *· *T;

2.    *i *:= 1; *j *:= 1;

3.    $A_{i}:=0̸;\;a_{i_{j}}:=0;$

4.    **for ***k *= 1 to *mB *+ 3*m*

5.      **if ***s*'[*k*] = T

6.       $A_{i}:=A_{i}\cup \left\{a_{i_{j}}\right\};$

7.       *j *+ +;

8.       **if ***s*[*k*] = T

9.        *i *+ +; *j *:= 1;

10.        $A_{i}:=0̸;$

11.       **end**

12.       $a_{i_{j}}:=0;$

13.      **else**

14.       $a_{i_{j}}++;$

15.      **end**

16.    **end**

It follows from the earlier discussions that $C_{\text{T}}\left(s^{\prime }\right)=C_{\text{T}}=\left\{{\text{G}}_{a_{i}}{\text{T}}_{0}\;:\;1\;\leq i\leq n\right\}$ and also that *s*' contains exactly 3*m − *1 base Ts. Furthermore, since *d*_*H *_(*s*, *s*') = 2*m*, we can deduce that *s*'[*k*] = *s*[*k*] if *s*[*k*] = T, ∀*k *∈ [1, *mB *+ 3*m − *1]. Notice that *s*[*k*] = T if and only if *k *can be written as a multiple of (*B *+ 3), that is, *k *= *i*(*B *+ 3) ∈ [1, *mB *+ 3*m − *1], ∀*i*. Therefore, *s*'[*k*] = T if *k *= *i*(*B *+ 3) ∈ [1, *mB *+ 3*m − *1], ∀*i*, which subsequently implies that $C_{\text{T}}\left(s^{\prime }\left[\left(i\;-\text{1}\right)\left(B+\text{ 3}\right)+\text{1,}\;\text{i(B}\;\text{ + }\;\text{3)}-1\right]\right)\subseteq C_{\text{T}}\left(s^{\prime }\right)$, for each *i *∈ [1, *m*]. Note that *s*[(*i − *1)(*B *+ 3) + 1, *i*(*B *+ 3) *− *1] is a substring of *s *that consists of (*B *+ 2) base Gs; it is located either strictly between two consecutive base Ts or strictly between one base T and one end of the string *s*. Since *C*_T_(*s^′^*[(*i − *1)(*B *+ 3) + 1, *i*(*B *+ 3) *− *1]) ⊆ *C*_T_(*s*'), we can let $C_{\text{T}}\left(s^{\prime }\left[\left(i-1\right)\left(B+3\right)+1,\;i\left(B+3\right)-1\right]\right)=\left\{{\text{G}}_{a_{i_{1}}}{\text{T}}_{0},\;{\text{G}}_{a_{i_{2}}}{\text{T}}_{0},\;\ldots ,\;{\text{G}}_{a_{i_{j}}}{\text{T}}_{0}\right\}$ such that $a_{i_{1}}+a_{i_{2}}+\cdots +a_{i_{j}}+j-1=B+2$. Since $\frac{B}{4}<a_{i_{j}}<\frac{B}{2}$, we can deduce that *j *= 3; hence $a_{i_{1}}+a_{i_{2}}+a_{i_{3}}=B$. Let$A_{i}=\left\{a_{i_{1}},\;a_{i_{2}},\;a_{i_{3}}\right\}$, for all *i *∈ [1, *m*]. Then, we can see that $A_{1},A_{2},...,A_{m}$ is a partition of A  such that the sum of integers in each subset is equal to *B*.

### Extensions to edit distance

Naturally we may extend our previous problem formulations to the edit distance (i.e., Levenshtein distance). The resulting two new problems are formally defined as follows.

**Definition 15 (The $\text{SNP - }\text{M}{\text{S}}_{P}$ problem) ***Given a string s and a collection of compomer spectra $C_{\sum }=\left\{C_{x}:x\in \Sigma \right\}$, find a string s*' *such that $C_{x}\left(s^{\prime }\right)\in C_{x}$, for all × *∈ Σ *and d_E _*(*s*, *s*') *is minimized*.

**Definition 16 (The $\text{SNP - M}{\text{S}}_{Q}$ problem) ***Given a string s and a collection of compomer spectra $C_{\sum }=\left\{C_{x}:x\in \Sigma \right\}$, find a string s*' *such that $C_{x}\subseteq C_{x}\left(s^{\prime }\right)$, for all *x∈∑*and d_E _*(*s*, *s*') *is minimized*.

These extensions make it possible to detect not only base substitutions but also base insertions and deletions. Hence, they would permit the mutation discovery in DNA sequences (see [1]). In the Additional file 1, we show that both $\text{SNP - }\text{M}{\text{S}}_{P}$ and $\text{SNP - M}{\text{S}}_{Q}$ are theoretically NP-hard, together with an exact dynamic programming algorithm for solving the $\text{SNP - }\text{M}{\text{S}}_{P}$ problem.

## Conclusions

To exploit the full potential of the SNP discovery approach using base-specific cleavage and mass spectrometry, in this paper we have studied two new combinatorial optimization problems, called $\text{SNP - }\text{M}{\text{S}}_{P}$ and $\text{SNP - M}{\text{S}}_{Q}$, respectively. We believe that any efficient solution to either problem could offer a more seamless integration of information in four complementary base-specific reactions than previously done in [1,2], thereby improving the capability of the underlying biotechnology (i.e., base-specific cleavage and mass spectrometry) for sensitive and accurate SNP discovery.

Although we cannot change the inherent complexity of our proposed dynamic programming algorithm for the $\text{SNP - }\text{M}{\text{S}}_{P}$ problem, we believe that by improving and optimizing its implementation, the compute runtime can be significantly reduced to the extent suitable for practical use. On the other hand, the NP-hardness result indicates that in the most general situation, solving the $\text{SNP - M}{\text{S}}_{Q}$ problem exactly in polynomial time is impossible unless P = NP. In more realistic situations where only a very few SNPs (e.g., two or three SNPs) occur in a target sample sequence, however, the problem can be quite easily tackled, e.g., using an exhaustive search approach. In the future work, we shall try to prove that the $\text{SNP - }\text{M}{\text{S}}_{P}$ problem is NP-hard and develop an efficient heuristic algorithm for the $\text{SNP - M}{\text{S}}_{Q}$ problem for practical use.

## Authors' contributions

XC conceived the study. All authors contributed to the problem analysis, read and approved the final manuscript.

## Competing interests

The authors declare that they have no competing interests.

## Supplementary Material

Additional file 1 **Extensions to edit distance**. The analysis results for the problems $\text{SNP - M}{\text{S}}_{P_{e}}$ and $\text{SNP - M}{\text{S}}_{Q_{e}}$ are presented. See "Additional file 1.pdf". Click here for file

## References

[B1] Bocker S SNP and mutation discovery using base-specific cleavage and MALDI-TOF mass spectrometry Bioinformatics 2003 19 Suppl 1 i44 53 10.1093/bioinformatics/btg1004 12855436

[B2] Krebs S Medugorac I Seichter D Forster M RNaseCut: a MALDI mass spectrometry-based method for SNP discovery Nucleic Acids Research 2003 31 7 10.1093/nar/gng037 PMC152822 12655025

[B3] Stanssens P Zabeau M Meersseman G Remes G Gansemans Y Storm N Hartmer R Honisch C Rodi CP Bocker S van den Boom D High-throughput MALDI-TOF discovery of genomic sequence polymorphisms Genome Research 2004 14 126 133 14707174 10.1101/gr.1692304 PMC314289

[B4] Hartmer R Storm N Bocker S Rodi CP Hillenkamp F Jurinke C van den Boom D RNase T1 mediated base-specific cleavage and MALDI-TOF MS for high-throughput comparative sequence analysis Nucleic Acids Research 2003 31 9 10.1093/nar/gng047 PMC154235 12711692

[B5] Honisch C Raghunathan A Cantor CR Palsson BO van den Boom D High-throughput mutation detection underlying adaptive evolution of Escherichia coli-K12 Genome Research 2004 14 12 2495 2502 10.1101/gr.2977704 15574828 PMC534674

[B6] RNaseCut webpage link http://www.vetmed.uni-muenchen.de/gen/forschung.html

[B7] Garey MR Johnson DS Complexity results for multiprocessor scheduling under resource constraints Siam Journal on Computing 1975 4 397 411 10.1137/0204035

[B8] Hulett H Will TG Woeginger GJ Multigraph realizations of degree sequences: Maximization is easy, minimization is hard Operations Research Letters 2008 36 5 594 596 10.1016/j.orl.2008.05.004

[B9] Bocker S Sequencing from compomers: Using mass spectrometry for DNA de novo sequencing of 200+ nt Journal of Computational Biology 2004 11 6 1110 1134 10.1089/cmb.2004.11.1110 15662201

[B10] Pevzner PA Tang HX Waterman MS An Eulerian path approach to DNA fragment assembly Proceedings of the National Academy of Sciences of the United States of America 2001 98 17 9748 9753 10.1073/pnas.171285098 11504945 PMC55524

