# Supplementary material for: Two combinatorial optimization problems for SNP discovery using base-specific cleavage and mass spectrometry
Source: BMC Syst Biol. 2012 Dec 12;6(Suppl 2):S5. doi: 10.1186/1752-0509-6-S2-S5 (PMC3521188; doi:10.1186/1752-0509-6-S2-S5)
Supplement: Additional file 1 — Extensions to edit distance. The analysis results for the problems SNP - MSPe and SNP - MSQe are presented. See "Additional file 1.pdf". [file 1752-0509-6-S2-S5-S1.pdf]

Additional Files

Additional file 1 — Extensions to edit distance

In what follows, we present the results for the problems SNP-MS$_{\mathcal{P}_e}$ and SNP-MS$_{\mathcal{Q}_e}$.

The NP-hardness of the SNP-MS$_{\mathcal{P}_e}$ problem

This section is dedicated to the proof of the NP-hardness of the SNP-MS$_{\mathcal{P}_e}$ problem. This proof is basically adapted from a proof presented in [1] to show the computational complexity of the *Sequencing From Compomers* problem. Let us start with a brief introduction of the well-known change-making problem.

Definition 1 (Change-making problem) *Given a set of positive integers $\mathcal{M} = \{m_1, m_2, \cdots, m_n\}$ and a positive integer $\tilde{m}$, do there exist nonnegative integers $\alpha_1, \alpha_2, \cdots, \alpha_n$ such that $\sum_{i=1}^{n} m_i \cdot \alpha_i = \tilde{m}$?*

The NP-completeness of the change-making problem was established in [2]. However, it is only weakly NP-complete as there exists a pseudo-polynomial algorithm to solve it [3]. The following lemma plays an important role in our proof of the NP-hardness of SNP-MS$_{\mathcal{P}_e}$.

Lemma 2 ([1]) *Let $F = \{2^e : e = 0, 1, \cdots, f\}$ for some positive integer f. For every integer j with $1 \leq j \leq 2^f$, there exist indices $\beta_1, \cdots, \beta_j \in F$ such that $\sum_{j'=1}^{j} \beta_{j'} = 2^f$.*

Theorem 3 *The SNP-MS$_{\mathcal{P}_e}$ problem is NP-hard when $|\Sigma| \geq 3$.*

Proof: To prove the hardness of the SNP-MS$_{\mathcal{P}_e}$ problem, we will make a polynomial reduction from the above change-making problem. Given an instance $\langle \mathcal{M}, \tilde{m} \rangle$ of the change-making problem, we define an instance $\langle s, \mathcal{C}_\Sigma \rangle$ of SNP-MS$_{\mathcal{P}_e}$ as follows:

- Let $f = \lceil \log_2 \tilde{m} \rceil$ and $F = \{2^e : e = 0, 1, \cdots, f\}$.
- Let $\Sigma = \{\mathrm{A}, \mathrm{G}, \mathrm{T}\}$ and $s = \epsilon$.
- Let $\mathcal{C}_\mathrm{A} = \{\mathrm{A}_0\mathrm{G}_\beta\mathrm{T}_0 : \forall \beta \in F\} \cup \{\mathrm{A}_0\mathrm{G}_0\mathrm{T}_0\}$, $\mathcal{C}_\mathrm{G} = \{\mathrm{A}_m\mathrm{G}_0\mathrm{T}_0 : \forall m \in \mathcal{M}\} \cup \{\mathrm{A}_0\mathrm{G}_0\mathrm{T}_0\}$ and $\mathcal{C}_\mathrm{T} = \{\mathrm{A}_{\tilde{m}}\mathrm{G}_{2^f}\mathrm{T}_0\}$ so that $\mathcal{C}_\Sigma = \{\mathcal{C}_\mathrm{A}, \mathcal{C}_\mathrm{G}, \mathcal{C}_\mathrm{T}\}$.

First, we need to check whether this reduction can be done in polynomial time in the size of an input instance $\langle \mathcal{M}, \tilde{m} \rangle$ of the change-making problem. With the natural binary encoding of integers, an input instance $\langle \mathcal{M}, \tilde{m} \rangle$ can be represented in $\Theta\left(\sum_{i=1}^{n} \log m_i + \log \tilde{m}\right)$ bits. In the first step of the above reduction, computing f and F take times in $O(\log \tilde{m})$ and $O(\log^2 \tilde{m})$, respectively. The second step can be easily done

in constant time. Note that the encoding of a compomer $A_iG_kT_l$ only needs $O(\log i + \log k + \log l)$ bits. Thus, the third step can be done in time $O\left(\sum_{i=1}^{n} \log m_i + \log^2 \tilde{m}\right)$. In total, the entire reduction would take only polynomial time in the instance size of $\langle \mathcal{M}, \tilde{m} \rangle$.

Next, we show that every feasible solution s'' to the reduced instance $\langle s, \mathcal{C}_\Sigma \rangle$ of SNP-MS$_{\mathcal{P}_e}$ is such that (i) s'' is a string of length $(\tilde{m} + 2^f)$ comprising exactly $\tilde{m}$ base As and 2^f base Gs, (ii) every maximal contiguous segment of s'' that contains only base As has length m for some $m \in \mathcal{M}$, and (iii) every maximal contiguous segment of s'' that contains only base Gs has length β for some $\beta \in F$. Note that the composition of base T is always zero in the compomer spectrum $\mathcal{C}_A$ with respect to the cut base A. Thus, no base T shall occur in any feasible solution s''. From the compomer spectrum $\mathcal{C}_T$ which contains only one compomer, we can know that s'' must comprise exactly $\tilde{m}$ base As and 2^f base Gs, thereby the length of s'' being $(\tilde{m} + 2^f)$. It further implies that every maximal contiguous segment of s'' that contains base As (resp. Gs) is a cleavage fragment with respect to the cut base G (resp. A). Therefore, the length of each such maximal contiguous segment shall be equal to the composition number of the corresponding base in its compomer, as we claimed in (ii) and (iii) above. Furthermore, since every feasible solution s'' is of length $(\tilde{m} + 2^f)$ while s is given as the empty string, we would always have $d_E(s, s'') = \tilde{m} + 2^f$. It turns out that, as long as s'' is a feasible solution, it is also an optimal solution to the reduced instance $\langle s, \mathcal{C}_\Sigma \rangle$ of SNP-MS$_{\mathcal{P}_e}$.

To complete the proof, we next show that there exist nonnegative integers $\alpha_1, \alpha_2, \cdots, \alpha_n$ such that $\sum_{i=1}^{n} m_i \cdot \alpha_i = \tilde{m}$ for the instance $\langle \mathcal{M}, \tilde{m} \rangle$ of the change-making problem if and only if there exists a feasible solution s' for the reduced instance $\langle s, \mathcal{C}_\Sigma \rangle$ of the SNP-MS$_{\mathcal{P}_e}$ problem.

Suppose that we have nonnegative integers $\alpha_1, \alpha_2, \cdots, \alpha_n$ such that $\sum_{i=1}^{n} m_i \cdot \alpha_i = \tilde{m}$. Clearly, $\sum_{i=1}^{n} \alpha_i \leq \tilde{m} \leq 2^f$ where $f = \lceil \log_2 \tilde{m} \rceil$. Then, we may use the following procedure to find the string s':

$s' := \emptyset$; $j := \sum_{i=1}^{n} \alpha_i$;
Compute indices $\beta_1, \cdots, \beta_j \in F$ such that $\sum_{j'=1}^{j} \beta_{j'} = 2^f$; // see Lemma 2
for $i = 1$ to n
  while $\alpha_i > 0$
    $s' += A^{m_i} G^{\beta_j}$; // append the string $A^{m_i} G^{\beta_j}$ to s'
    $\alpha_i - -$; $j - -$;
  end
end

As one can easily check, the resulting string s' contains exactly $\tilde{m}$ base As and 2^f base Gs with $\mathcal{C}_{\mathrm{A}}(s') \subseteq \mathcal{C}_{\mathrm{A}}$ and $\mathcal{C}_{\mathrm{G}}(s') \subseteq \mathcal{C}_{\mathrm{G}}$. Therefore, s' is a feasible solution and hence an optimal solution as well to the reduced instance $\langle s, \mathcal{C}_\Sigma \rangle$ of the SNP-MS$_{\mathcal{P}_e}$ problem.

Conversely, suppose that the string s' is an optimal solution to the reduced instance $\langle s, \mathcal{C}_\Sigma \rangle$ of the SNP-MS$_{\mathcal{P}_e}$ problem. As a result, s' must contain exactly $\tilde{m}$ base As and 2^f base Gs. Let α_i be the number of cleavage fragments of size m_i when the string s' is cleaved with respect to the cut base G. Note that each (nonempty) cleavage fragment with respect to the cut base G is a maximal contiguous segment of s' that comprises only base As. Hence, its size must be equal to some integer value m_i in $\mathcal{M}$ because $\mathcal{C}_{\mathrm{G}}(s') \subseteq \mathcal{C}_{\mathrm{G}}$. With these observations, one can further check that all the α_i values found above are nonnegative integers such that $\sum_{i=1}^{n} m_i \cdot \alpha_i = \tilde{m}$. We now reach the conclusion that the SNP-MS$_{\mathcal{P}_e}$ problem is NP-hard when $|\Sigma| \geq 3$. ■

The NP-hardness of the SNP-MS$_{\mathcal{Q}_e}$ problem

In this section, we shall show the NP-hardness of the SNP-MS$_{\mathcal{Q}_e}$ problem in a quite similar way to proving the NP-hardness of the SNP-MS$_{\mathcal{Q}}$ problem.

Theorem 4 *The SNP-MS$_{\mathcal{Q}_e}$ problem is NP-hard, even when $|\Sigma| = 2$.*

Proof: We prove it by a polynomial reduction from the restricted variation of the 3-partition problem (see Definition **??**). As an input for the 3-partition problem, we are given a set of distinct integers $\mathcal{A} = \{a_1, a_2, \cdots, a_n\}$, where $n = 3m$, $\sum_{i=1}^{n} a_i = mB$, and $\frac{B}{4} < a_i < \frac{B}{2}$, $\forall 1 \leq i \leq n$. Then we construct an instance $\langle s, \mathcal{C}_\Sigma \rangle$ of the SNP-MS$_{\mathcal{Q}_e}$ problem as follows:

- Let $\Sigma = \{\mathrm{G}, \mathrm{T}\}$.
- Let s be the string such that $s \cdot \mathrm{T} := (\mathrm{G}^B\mathrm{T})^m$. That is, let $s \cdot \mathrm{T}$ be the concatenation of m copies of the fragment $\mathrm{G}^B\mathrm{T}$, where G^B denotes the concatenation of B consecutive base Gs. Note that $|s| = m(B+1) - 1 = mB + m - 1$ and there are $(m-1)$ base Ts in s.
- Let $\mathcal{C}_{\mathrm{G}} := \{\mathrm{G}_0\mathrm{T}_0, \mathrm{G}_0\mathrm{T}_1\}$ and $\mathcal{C}_{\mathrm{T}} := \{\mathrm{G}_{a_i}\mathrm{T}_0 : 1 \leq i \leq n\}$, so that $\mathcal{C}_\Sigma = \{\mathcal{C}_{\mathrm{G}}, \mathcal{C}_{\mathrm{T}}\}$.

Using the same arguments as in Section "*The NP-hardness of SNP-MS$_{\mathcal{Q}}$*", one can easily check that the above construction can be done in polynomial time in the size of the input instance of the 3-partition problem

(where the integers are encoded in unary rather than binary). Furthermore, every feasible solution s'' to the reduced instance $\langle s, \mathcal{C}_\Sigma \rangle$ of the SNP-MS$_{\mathcal{Q}_e}$ problem is such that (i) s'' contains at least mB base Gs and at least $3m-1$ base Ts and (ii) $d_E(s,s') \geq |s'| - |s| \geq (mB+3m-1)-(mB+m-1) = 2m$.

Next, we shall show that there exists a valid partition for the input instance $\langle \mathcal{A}, B \rangle$ of the 3-partition problem if and only if there exists an optimal solution s' for the reduced instance of the SNP-MS$_{\mathcal{Q}_e}$ problem such that $d_E(s,s') = 2m$.

Suppose that $\mathcal{A}$ can be partitioned into m subsets $\mathcal{A}_1, \mathcal{A}_2, \ldots, \mathcal{A}_m$ such that, for each subset $\mathcal{A}_i = \{a_{i_1}, a_{i_2}, a_{i_3}\}$, its size is three and its integer elements adds to exactly B, that is, $|\mathcal{A}_i| = 3$ and $\sum_{j=1}^{3} a_{i_j} = B$, $\forall 1 \leq i \leq m$. Then, we may use the same procedure as the one used in the proof of Theorem **??** to find the string s'. As we already know from there, the constructed string s' is such that $|s'| = mB + 3m - 1$, $\mathcal{C}_\mathrm{G} \subseteq \mathcal{C}_\mathrm{G}(s')$, and $\mathcal{C}_\mathrm{T} \subseteq \mathcal{C}_\mathrm{T}(s')$. Therefore, s' is a feasible solution to the reduced instance $\langle s, \mathcal{C}_\Sigma \rangle$ of the SNP-MS$_{\mathcal{Q}_e}$ problem with $d_E(s,s') \geq 2m$. To find $d_E(s,s')$, we focus on aligning each substring $\mathrm{G}^{a_{i_1}}\mathrm{T}\mathrm{G}^{a_{i_2}}\mathrm{T}\mathrm{G}^{a_{i_3}}\mathrm{T}$ of the string s' with a fragment $\mathrm{G}^B\mathrm{T}$ of the string s. Since $\sum_{j=1}^{3} a_{i_j} = B$, $\forall 1 \leq i \leq m$, we can find that each such alignment can be achieved with two gaps. In other words, the edit distance between $\mathrm{G}^{a_{i_1}}\mathrm{T}\mathrm{G}^{a_{i_2}}\mathrm{T}\mathrm{G}^{a_{i_3}}\mathrm{T}$ and $\mathrm{G}^B\mathrm{T}$ is no more than 2, which further implies that $d_E(s,s') \leq 2m$ as there are m such alignments that can be seen between s and s'. Therefore, we can conclude that s' is indeed an optimal solution to the reduced instance $\langle s, \mathcal{C}_\Sigma \rangle$ of the SNP-MS$_{\mathcal{Q}_e}$ problem.

Conversely, suppose that the string s' is an optimal solution to the reduced instance $\langle s, \mathcal{C}_\Sigma \rangle$ of the SNP-MS$_\mathcal{Q}$ problem such that $d_E(s,s') = 2m$. Note that there are at least $3m-1$ base Ts in s' but only $m-1$ base Ts in s. As a result, the alignment between s and s' must contain $2m$ gaps from s, each of which is mapped to a base T from s'. If all these gaps in s are substituted by base Gs, then we may use the same procedure as the one used in the proof of Theorem **??** to find a valid partition for the input instance $\langle \mathcal{A}, B \rangle$ of the 3-partition problem. This concludes our proof. ■

An exact dynamic programming algorithm for the SNP-MS$_{\mathcal{P}_e}$ problem

In this section, we present an exact dynamic programming algorithm for solving the SNP-MS$_{\mathcal{P}_e}$ problem. This algorithm is developed in the same spirit as the dynamic programming algorithm for solving the SNP-MS$_\mathcal{P}$ problem in Section "*An exact dynamic programming algorithm for SNP-MS$_\mathcal{P}$*". As the SNP-MS$_\mathcal{P}$ problem is aimed to minimize Hamming distance, the optimal solution string s' shall have the same length as the input reference string s. However, for the SNP-MS$_{\mathcal{P}_e}$ problem which is aimed to minimize the edit

distance, we cannot know in advance the exact length of the optimal solution string s'. Therefore, we expect that solving the SNP-MS$_{\mathcal{P}_e}$ problem would become more challenging than solving SNP-MS$_{\mathcal{P}}$.

Recall that the input to the SNP-MS$_{\mathcal{P}_e}$ problem is a reference string s and a collection of compomer spectra $\mathcal{C}_\Sigma$, and the desired output is a string s' such that $\mathcal{C}_x(s') \subseteq \mathcal{C}_x$, for all $x \in \Sigma$ and $d_E(s, s')$ is minimized. As in Section "*An exact dynamic programming algorithm for SNP-MS$_{\mathcal{P}}$*", we once again assume that all the bases in the alphabet Σ will eventually appear in the optimal solution to a given instance of the SNP-MS$_{\mathcal{P}_e}$ problem. In addition, most definitions and notations in that section still apply here, unless otherwise stated.

Given a string $t \in \mathcal{I}_x$, let $\mathcal{E}(i, t)$ denote the minimum edit distance between the prefix of s of length i and a string which is such that

- all its substrings are I-compatible with $\mathcal{C}_\Sigma$,
- it begins with a string from $\mathcal{L}_y$ for some $y \in \Sigma$, and
- it ends with the given string t.

To compute $\mathcal{E}(i, t)$, as already done in Section "*An exact dynamic programming algorithm for SNP-MS$_{\mathcal{P}}$*", we first find in the string $x \cdot t$ the rightmost position k at which the base $(x \cdot t)[k]$ is its first occurrence. Formally, we may write

$$k = \max \Big\{ j : \forall i, 1 \le i < j \le |x \cdot t|, (x \cdot t)[i] \ne (x \cdot t)[j] \Big\}.$$

Then, let $x' := (x \cdot t)[k]$, $p := (x \cdot t)[1, k-1]$, and $q := (x \cdot t)[k, |x \cdot t|]$. Note that $x' \ne x$ and the string p contains all the bases of Σ except x'. Finally, we apply the following recurrence relation to compute $\mathcal{E}(i, t)$:

$$\mathcal{E}(i, t) = \min_{\substack{0 \le j \le i \\ t' \in \mathcal{I}_{x'} \\ \exists t'', t' = t'' \cdot p}} \Big\{ \mathcal{E}(i - j, t') + d_E(s[i - j + 1, i], q) \Big\},$$

with the initial conditions $\mathcal{E}(0, t) = |t|$, for all $t \in \mathcal{I}_x$ and $x \in \Sigma$. We let $s[i - j + 1, i] = \epsilon$ in case $j = 0$. Note that the above minimization is taken over all those strings t' in $\mathcal{I}_{x'}$ which have p as the suffix. If there is no such a string in $\mathcal{I}_{x'}$, then we let $\mathcal{E}(i, t) = \infty$.

Theorem 5 *Let s' be the string that leads to*

$$d_E(s, s') = \min_{\forall t \in \mathcal{R}_x, x \in \Sigma} \mathcal{E}(|s|, t),$$

then s' would be an optimal solution to the input instance $\langle s, \mathcal{C}_\Sigma \rangle$ of the SNP-MS$_{\mathcal{P}_e}$ problem.

Proof: The correctness of the above dynamic programming algorithm can be established in the same spirit as for the dynamic programming algorithm presented in Section "*An exact dynamic programming algorithm for SNP-MS*$_{\mathcal{P}}$". Here, we omit its lengthy proof. ∎

We can also have the following corollary.

Corollary 6 *The above dynamic programming algorithm can solve the SNP-MS*$_{\mathcal{P}_e}$ *problem in polynomial time when* $|\Sigma| = 2$.

References

1. Bocker S: **Sequencing from compomers: Using mass spectrometry for DNA de novo sequencing of 200+ nt**. *Journal of Computational Biology* 2004, **11**(6):1110–1134.

2. Lueker GS: **Two NP-complete problems in nonnegative integer programming**. Tech. Rep. TR-178, Department of Electrical Engineering, Princeton University 1975.

3. Wright JW: **Change-Making Problem**. *Journal of the ACM* 1975, **22**:125–128.
